# High-Performance Regenerated Silk Fibers as Building Blocks of Tissue Scaffolds: The European THOR Project

**DOI:** 10.3390/biomimetics11080566

**Published:** 2026-08-08

**Authors:** José Pérez-Rigueiro, Atocha Guedán-Durán, Fivos Panetsos, Gianna Arencibia, Gustavo V. Guinea, Luis Colchero, Miriam Quero, Jaime Espinosa, Alessandro Rizzi, Tando Maduna, Anna Pancho, Marsela Hakani, Andreas Vlachos, Julia Sepúlveda-Díaz, Alan Morin, Michele Papa, Giovanni Cirillo, Assunta Virtuoso, Ciro De Luca

**Affiliations:** 1 Bioactive Surfaces S.L., C/Puerto de Navacerrada 18, 28260 Galapagar, Spain; 2 Centro de Tecnología Biomédica (CTB), Universidad Politécnica de Madrid, 28223 Pozuelo de Alarcón, Spainalessandro.rizzi.8@studenti.unipd.it (A.R.); 3 Departamento de Ciencia de Materiales, ETSI Caminos, Canales y Puertos, Universidad Politécnica de Madrid, 28040 Madrid, Spain; 4 Centro de Investigación Biomédica en Red de Bioingeniería, Biomateriales y Nanomedicina (CIBER-BBN), Instituto de Salud Carlos III, 28029 Madrid, Spain; 5 Biomaterials and Regenerative Medicine Group, Instituto de Investigación Sanitaria del Hospital Clínico San Carlos (IdISSC), 28040 Madrid, Spain; 6 Department of Neuroanatomy, Institute of Anatomy and Cell Biology, Faculty of Medicine, University of Freiburg, 79104 Freiburg, Germany; 7 Microfluidics Innovation Center S.A.S., 75011 Paris, France; 8 SYSBIO Centre of Systems Biology, University of Milano-Bicocca, 20126 Milano, Italy; 9 Laboratory of Neuronal Networks Morphology and Systems Biology, Department of Mental, Physical Health and Preventive Medicine, University of Campania “Luigi Vanvitelli”, 80138 Naples, Italyassunta.virtuoso@unicampania.it (A.V.);

**Keywords:** regenerated silk, spinning, tissue engineering, endothelial cell, organotypic slice

## Abstract

The European Pathfinder THOR project envisages the creation of a vascularized fragment of tissue that can be implanted in a patient using regenerated silk fibers as its building blocks. The selection of regenerated silk as the main building block of the scaffold relies heavily on its outstanding biocompatibility in comparison with either other artificial polymeric fibers or even natural silk fibers. Additionally, regenerated fibers produced through the Dynamic Dope Destabilization Spinning (D3S^TM^) process are shown to exhibit high mechanical performance as reflected in values of strain at breaking and work to fracture comparable to those of the natural material. It is further shown that these fibers are endowed with the unique property of self-adhesion whereby hydrated fibers attach to one another and may sustain detachment forces of up to a few tens of MPa, a property that facilitates the generation of the scaffold with the fibers as its basic building block. Lastly, regenerated silk fibers are shown to be efficiently decorated with either peptides or small proteins, such as the vascular endothelial growth factor (VEGF), or with antibodies. The performance of both non-functionalized and decorated silk fibers is assessed in two different *in vitro* biological systems: (1) endothelial cell cultures, and (2) organotypic brain slice cultures. Together, these results support the use of regenerated silk fibers as versatile building blocks for biofunctional tissue scaffolds and provide experimental validation of the tissue engineering strategy established by the THOR project.

## 1. Introduction

Tens of millions of people with organ failure or suffering from degenerative diseases are waiting for a novel cellular therapy or for a transplant of a donor-compatible organ. Unfortunately, a great majority of these patients will face a critical situation while identifying and receiving the compatible tissue or organ they need. In this regard, and despite significant advances in tissue engineering, not a single artificial tissue has been used to replace a part of an organ, with the exception of simple or avascular tissues like skin or cartilage [1]. Although the whole project of building a fully engineered tissue or organ presents significant technical hurdles [2], two main impediments stand out: (1) the impossibility to engineer physiological vascular networks to provide O_2_ and nutrients to the artificial tissue [3], and (2) the difficulty in the formulation of suitable bioinks that can be used in the 3D bioprinting of the target tissues and organs [4,5,6]. These impediments are exponentially amplified when attempting to model or reconstruct neural tissues, where the brain vasculature forms an exceptionally specialized architectural and functional barrier.

Within the central nervous system (CNS), the microvasculature does not simply serve as a conduit for passive perfusion; instead, it aggregates with astrocytes, pericytes, and microglia to construct the neurovascular unit (NVU) and maintain the highly restrictive blood–brain barrier (BBB). Endothelial cells within the brain vasculature display unique phenotypic properties, including specialized continuous tight junctions (e.g., claudin-5, occludin) and highly regulated polarized efflux transporters that govern metabolic homeostasis and shield neural tissue from systemic fluctuations. Replicating this intricate, shear-sensitive cerebral microenvironment remains an unresolved bottleneck in neurovascular tissue engineering [7,8]. In this context, conventional tissue engineering approaches, including bioprinting, decellularization and electrospinning, among others, tend to yield largely disorganized structures with a poor degree of specialization. Specifically, these constructs lack an oriented vascular geometry able to provide O_2_ and nutrients to the cells, which limits the size of the artificial tissue to a maximum dimension of approx. 200 μm [9,10], an effect that is widely known as the “necrotic core” in the field of organoid culture [11,12,13].

**Figure 1:**
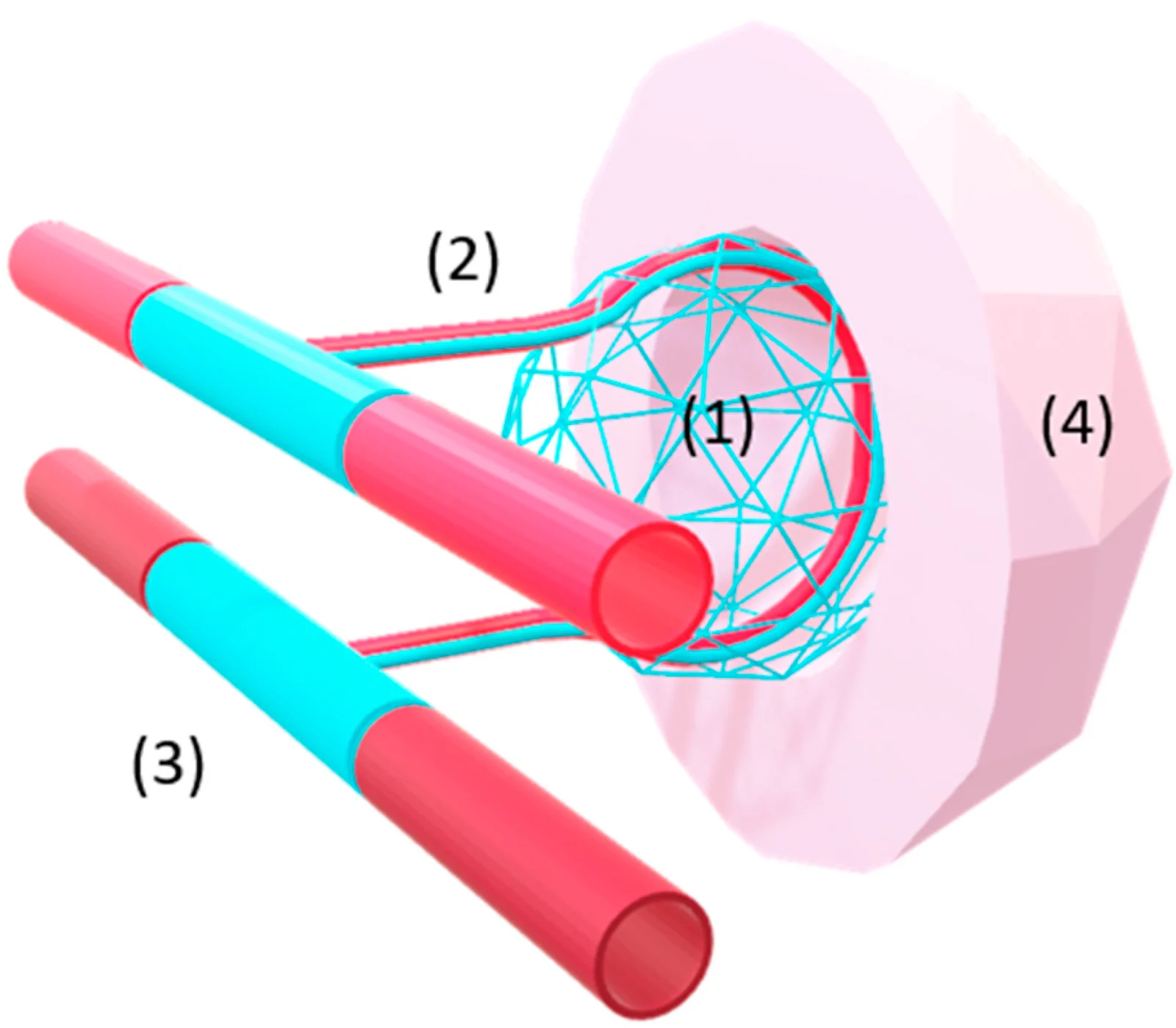
Graphical summary and essential elements of the Web Weaving Technology (WWT). (1) regenerated silk fibrillary scaffold, (2) guided blood capillary system, and (3) artificial vessels connected to the patient’s vasculature. The fibrillary scaffold is directly in contact with the functional tissue of the patient (4).

In this context, the European Pathfinder THOR project is intended to overcome the drawbacks brought up above by developing a novel tissue engineering technology, termed Web Weaving Technology (WWT), that might produce essentially any type of human tissue to be subsequently implanted in the patient (Figure 1).

In this regard, the creation of such an engineered tissue and, in particular, of the necessary microvasculature requires the usage of structural guides capable of recruiting and physically aligning both brain-specific endothelial cells and also the rest of the cells of the neurovascular unit.

In this work, we are going to show how regenerated silk fibers are ideally suited to become the building blocks of the scaffold due to their combination of outstanding biocompatibility [14,15,16], mechanical high performance [17] and the possibility of being efficiently decorated with bioactive molecules [18]. The combination of these three characteristics is hardly found in any other material, either natural or artificial. In this regard, we shall concentrate below on those aspects directly related to the role of regenerated silk fibers as building blocks of the fibrillary scaffold and, consequently, as the basis of the WWT.

Following this rationale, we present below the main aspects related to the regenerated silk fibers employed as the building blocks of the tissue scaffolds in the WWT as developed within the framework of the THOR project (https://thor-project.eu/en/ (accessed on 30 June 2026)). Thus, the Dynamic Dope Destabilization Spinning (D3S^TM^) process used for the production of the fibers is described and complemented with the mechanical characterization of the material. Subsequently, the self-adhesion ability of regenerated silk fibers, a key finding of the THOR project, is presented and characterized. This new property of silk fibers is essential for the development of the WWT, since it provides a straightforward procedure to bind the fibers that must create the 3D fibrillary structure. Crucially, to meet the complex biological demands of the neurovascular interface, two different decoration strategies are discussed and validated based either on the usage of small extracellular matrix-derived peptides or growth factors (such as IKVAV and VEGF), or on antibodies specific to the endothelial lineage (anti-CD31). The performance of the material when combined with the biological system is first tested with in vitro cell cultures of immortalized endothelial cells and primary mouse embryonic brain endothelial cells (MEBECs), establishing a robust baseline for a truly guided, BBB-emulating capillary network within the tissue scaffold. Subsequently, the performance of the material in connection with living neural boundaries is further assessed using organotypic brain slices as a preliminary step for the characterization of the structural and metabolic interaction between the engineered scaffold and complex biological systems.

## 2. Materials and Methods

### 2.1. Spinning of Silk Regenerated Fibers Through the D3S^TM^ Process

The Dynamic Dope Destabilization Spinning (D3S^TM^) is a biomimetic process that allows the spinning of high-performance regenerated silkworm silk fibers from a fibroin solution. The D3S^TM^ starts with the degumming for 30 min of the silkworm (*Bombyx mori*) cocoons at a proportion of 1% (*w*/*v*). The degumming solution consists of water with 0.1% (*w*/*v*) Na_2_CO_3_. The cocoons are washed in distilled water (1 L per 6 g of pristine cocoons) and stirred gently for 5 min. The washing of the silk is repeated a total of five times. After washing, the fibroin fibers are spread on aluminum foil and dried inside an oven at 30 °C overnight.

The fibroin solution is obtained by dissolving the fibroin fibers in a 9.3 M LiBr solution. The volume of the LiBr solution is determined under the condition that the weight of the LiBr salt is five times that of the dry weight of the fibroin fibers. The silk solution in LiBr is heated at 65 °C for four hours. In order to remove the LiBr, the fibroin solution is introduced into 3500 MW cutoff dialysis tubing, and dialysis proceeds against 2 L of distilled water. Water is replaced twice a day for three days. Lastly, the dialysed fibroin solution with a concentration of approx. 4% (*w*/*v*) is subjected to a reverse dialysis process in order to reach the desired fibroin concentration. Reverse dialysis proceeds for 16 h against a distilled water solution containing 25% (*w*/*v*) Polyethyleneglycol of MW = 8000 Da (PEG 8000; Thermofisher, Waltham, MA, USA) and 1 M CaCl_2_. The resulting fibroin solution (dope) corresponds to a concentration of 25% (*w*/*v*) in the 1 M CaCl_2_ aqueous solution.

The processing parameters of D3S^TM^ can be broken down into three sets: chemical, geometrical, and physical. The chemical parameters comprise the composition of the dope, the composition of the focusing fluid and the composition of the coagulating bath. The composition of the dope consists of the 25% aqueous fibroin solution (*w*/*v*) whose preparation has been described in detail above. The spinning of the fibers used in this work used the same composition for the focusing fluid and for the coagulating bath: 80% ethanol/20% 1 M acetic acid in water. The geometrical parameters include the distance between the end of the capillary, d, the tapering angle of the capillary, α, and the diameter of the nozzle outlet, D. The values used for these geometric parameters were: d= 1700 μm, α = 90° and D = 400 μm. Lastly, the physical parameters comprise the dope flow rate, Q_d_, the focusing fluid flow rate, Q_f_, the take-up speed, V_tu_, the post-spinning drawing speed, V_PS_, and the temperatures of the dope, T_d_, of the focusing fluid, T_f_, and of the coagulating bath, T_c_. The set of values used for the preparation of the fibers were: Q_d_ = 200 μL/h, Q_f_ = 160 mL/h, V_tu_ = 8.6 cm/s, V_PS_ = 11.2 cm/s, T_d_ = 20 °C, and T_f_ = T_c_ = 30 °C.

### 2.2. Mechanical Characterization of the Silk Regenerated Fibers

Tensile tests of the regenerated fibers in air were performed as described elsewhere [17]. Briefly, regenerated silk fibers were mounted on aluminum foil frames with a base length of 20 mm. A tensile testing machine, Instron 4411 (Norwood, MA, USA), was used for tensile testing at a displacement rate of 1 mm/min and nominal environmental conditions of 25 °C and 50% RH. The displacement of the crosshead (resolution ± 10 μm) was taken directly for the calculation of strain, since the stiffness of the experimental setup is estimated to be 1000 times larger than that of the fiber. The force exerted on the fiber was measured with a balance Precisa 520 PT 220 A (resolution ± 1 μN; Precisa Gravimetrics AG, Zurich, Switzerland). The cross-sectional area of the fibers was determined from optical micrographs using a Leica EZ4 HD microscope (Leica microsystems, Wetzlar, Germany), although some samples were selected for observation in an SEM Hitachi S-3000-N (Hitachi High-Tech Corporation, Tokyo, Japan) at a voltage of 10 kV. Previous to SEM observation, the samples were sputter-coated with gold for 2 min (Q150R Plus, Quorum Technologies Ltd., Laughton, UK). Tensile tests in water were performed under the same conditions, except that the fibers were immersed in water for at least 30 min before starting the test and remained submerged in water until its ending. Some fibers were subjected to wet stretching, a process that consists of stretching the fiber while immersed in water up to a given percentage of its initial length, and subsequent drying in air prior to tensile testing.

### 2.3. Self-Adhesion Test of Regenerated Silk Fibers

**Figure 2:**
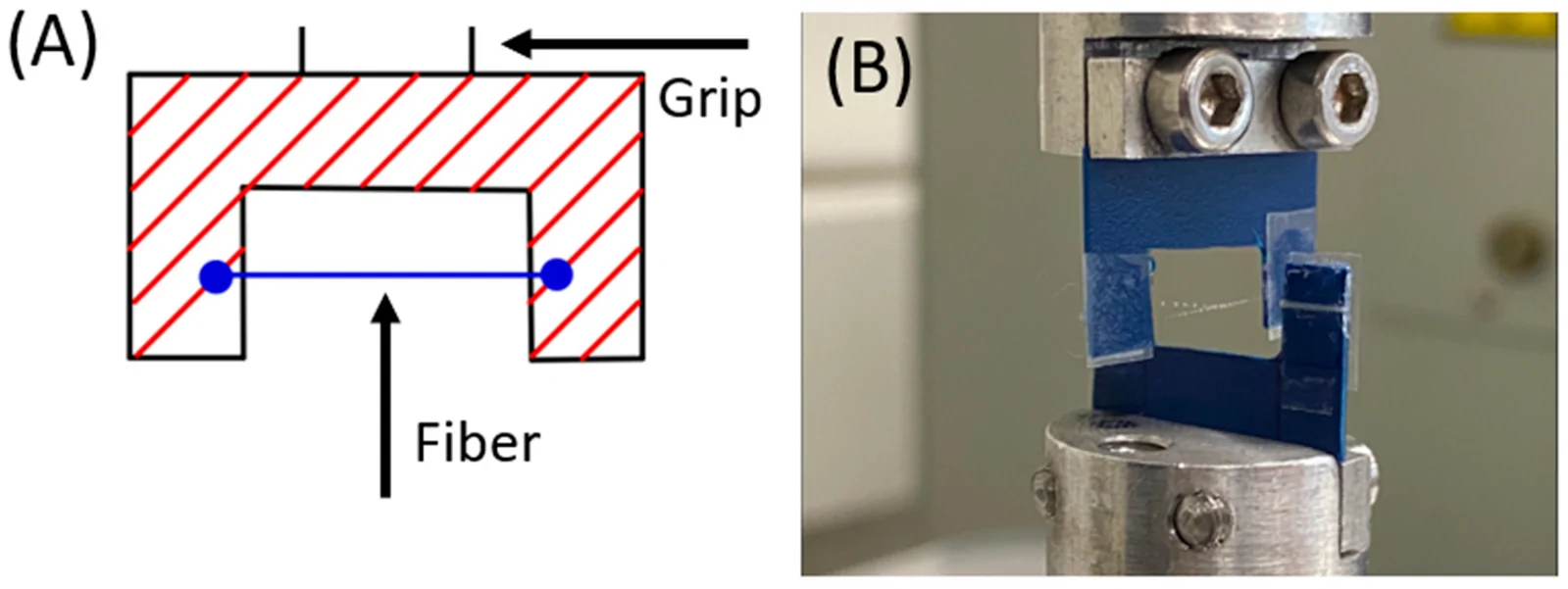
(**A**) Sample mounting for the adhesion test. (**B**) Geometry of the experimental setup for the self-adhesion test.

The mounting of the silk fiber for the adhesion test is presented in Figure 2A. As shown in this Figure, the fiber is glued at the ends of a U-shaped plastic frame with a span length of 15 mm and two of these frames are fixed to the upper and lower grips of the tensile testing machine, so that the fibers are placed at an angle of 90° (Figure 2B). The adhesion test proceeds in four steps: (i) contact, (ii) pushing, (iii) pulling, and (iv) detachment. The experimental parameters that define the self-adhesion test are the speed of approach and detachment, the pushing force, the duration of the contact step, as well as the hydration and drying steps. Adhesion tests were performed in an Instron 4411 tensile testing machine using a balance Precisa 520 PT 220A (resolution 1 μN) for the measurement of the forces. The approach and detachment steps proceeded at a speed of 1 mm/min. A humidifier was used for the hydration steps.

### 2.4. Decoration of the Fibers with the IKVAV Peptide and the VEGF Growth Factor

The decoration of the fibers with the IKVAV peptide and with the vascular endothelial growth factor (VEGF) employed the 1-Ethyl-3-(3-dimethylaminopropyl)carbodiimide/N-hydroxysuccinimide (EDC/NHS) crosslinking chemistry as described elsewhere [18]. Briefly, a solution of EDC (0.5 mg/mL) and NHS (0.13 mg/mL) in 0.1 M 2-(N-morpholine)-ethanesulfonic acid (MES) at pH = 5.5 was prepared. 50 μL of the EDC/NHS solution was added to each glass coverslip with the fibers on its surface and incubated for 15 min. The stock solutions of the IKVAV peptide and VEGF were prepared at a concentration of 170 μg/mL (IKVAV) and 0.7 μg/mL (VEGF) in 0.1 MES buffer, and 150 μL of the stock solution was added to each coverslip. The samples were incubated for 4 h at room temperature and washed twice with PBS (phosphate-buffered saline) for 1 h. In order to verify the covalent immobilization of the bioactive molecules to the fibers, two batches of Fluorescein isothiocyanate (FITC)-derivatized IKVAV and VEGF were prepared, and the decorated fibers were observed in a fluorescence microscope. FITC-derivatization of both IKVAV and VEGF proceeded by first dissolving each molecule in PBS at a concentration of 500 μg/mL. Subsequently, FITC dissolved in PBS was added up to a final concentration of 250 μg/mL (peptide/VEGF:FITC ratio in weight 1:0.5). The peptide/VEGF-FITC solutions were incubated at room temperature and protected from light for 3 h. In order to eliminate the excess of FITC molecules, the fibers were dialyzed using Spectra/Por Cellulose Ester Dialysis Tubing (molecular weight cut-off 500 Da) against PBS. The dialysis medium was changed every 12 h for a period of 36–48 h. Before each medium change, the absorbance of 1 mL of the PBS dialysis medium at a wavelength of 495 nm was measured in a UV/visible spectrophotometer (Halo Rb10, Dynamica Scientific Ltd., Livingston, UK) and compared with a FITC calibration curve to calculate the number of free molecules in the solution. It was verified that absorbance was below the resolution of the spectrophotometer after the last dialysis step. Fluorescence was observed with a DMI3000B Inverted Leica DMIRB (Leica microsystems, Wetzlar, Germany) equipped with a Leica DC100 digital camera (Leica microsystems, Wetzlar, Germany). Observation conditions: exposure time = 1.3 ms, gain = 2.1 x and gamma = 0.68. VEGF was used in the present study as a proangiogenic and endothelial bioactive cue. Antimicrobial properties of either soluble VEGF or VEGF immobilized on the regenerated silk fibers were not evaluated. Accordingly, no bacterial challenge, growth-inhibition, bacterial viability, adhesion, or biofilm assays were performed.

### 2.5. Decoration of the Fibers with Biotinylated Anti-CD31 Antibody

A 50 μL droplet of a sulfo-NHS-LC-biotin solution (0.5 mg/mL sulfo-NHS-LC-biotin in PBS, pH 7.4) was deposited on each coverslip and incubated for 2 h at 4 °C under sterile conditions. After incubation, the coverslips were washed with 100 μL of sterile PBS solution for 5 min (×3) and 100 μL of sterile distilled water (×1). After completing the washing, 50 μL of a 0.2 mg/mL streptavidin (from *Streptomyces avidinii*) solution in PBS (pH = 7.4) was added and incubated for 1 h at 4 °C. The coverslips were washed with 100 μL of sterile PBS for 5 min (×3). 50 μL of a 10 μg/mL anti-CD31 biotinylated antibody in PBS was added and incubated for 1 h at 4 °C in the dark. Lastly, the coverslips were washed with 100 μL of sterile PBS for 5 min (×3).

### 2.6. Claudin-5 Expression and Cell Alignment on Regenerated Silk Fibers with Endothelial Cell Cultures

Mouse embryonic brain-endothelial cells (MEBECs) were purchased from Cellbiologics, Inc. (2201 West Campbell Park Drive, Chicago, IL 60612, USA) and cultivated with the recommended endothelial basal medium, supplemented with 0.1% hydrocortisone, 0.1% Epithelial growth factor (EGF), 0.1% VEGF, 0.1%, Endothelial cell growth supplement (ECGS), 0.1% Heparin, 5% Fetal bovine serum (FBS), and penicillin/streptomycin at 37 °C in an atmosphere of 5% CO_2_. Before cell seeding, scaffolds were sterilized by autoclaving and kept in sterile PBS. MEBECs were then seeded onto the scaffolds at a density of 1 × 10^4^ cells per well.

MEBEC morphology and degree of alignment were assessed 5 days in vitro (DIV5) by using image analysis of immunofluorescence (IF)-stained cells. This time point was chosen based on preliminary data showing cell confluence at 5 days for the seeding density used. Briefly, scaffolds at DIV5 were fixed in 4% PFA (paraformaldehyde) and then washed with PBS. Immunostaining was performed on each scaffold and on control coverslips. Fluorescence was imaged using a Zeiss LSM-900 microscope (Carl Zeiss AG, Carl Zeiss Jena GmbH, Oberkochen, Germany). Images were then analyzed with Fiji software (Fiji Stable, National Institutes of Health, Bethesda, MD, USA). To precisely characterize cell alignment relative to the scaffold architecture, images were pre-processed by orienting the linear silk fibers perpendicular to the horizontal baseline of the image frame. Specific ROIs (Regions of Interest) were then selected within the interstitial spaces bounded by two adjacent linear silk fibers, ensuring the fibers themselves were strictly excluded from the analysis area to prevent background or autofluorescence artifacts. The degree of cell alignment within these defined inter-fiber spaces was subsequently calculated using the Directionality tool in Fiji. The tool was executed using the Fourier components method, which applies a Fast Fourier Transform (FFT) to convert the spatial image data into a frequency domain map, breaking down the structures into their angular components. The software generates a directionality histogram based on this power spectrum, which is then fitted with a Gaussian curve. From this mathematical fit, the preferred orientation and the dispersion were determined. Claudin-5 immunofluorescence was quantified in Fiji as integrated fluorescence (IF) density, abbreviated as DxA, and calculated as the mean fluorescence intensity within the selected region of interest multiplied by the ROI area. The same ROI area was analyzed for all experimental groups. Because fluorescence intensity is dependent on the image-acquisition and analysis conditions, the resulting DxA values are reported in arbitrary units (a.u.). Three images were analyzed per group (*n* = 3 images). All images used for quantitative comparison were acquired using an identical microscope, laser power, detector gain, exposure, and image-processing settings. The expression and organization of tight junctions were visualized by immunostaining MEBECs for claudin-5 on scaffolds at DIV5. Briefly, all samples were fixed in 4% PFA (EMD Chemicals, Gibbstown, NJ, USA) for 30 min and washed with PBS 3 times for 5 min each. Next, cells were permeabilized by incubation in 0.1% Triton X-100 (Sigma-Aldrich, Waltham, MA, USA) for 10 min, washed 3 times with PBS for 5 min each, and then incubated in 1% sheep serum (Santa Cruz Biotechnology, Inc., Santa Cruz, CA, USA) for 30 min to reduce non-specific background staining. The samples were incubated for 2 h with a primary antibody against claudin-5 (Ab 4C3C2 Invitrogen, Waltham, MA, USA), followed by a 1 h incubation in a secondary antibody at room temperature. All samples were counter-stained using Vectashield with 4′,6-diamidino-2-phenylindole (DAPI, Vector Laboratories, Burlingame, CA, USA) to visualize cell nuclei and imaged using a Zeiss LSM-900 microscope. All results are reported as the mean ± standard deviation. Statistical analysis utilized a one-way analysis of variance (ANOVA), with *p* ≤ 0.05 set as the criterion for statistical significance, followed by Tukey’s post hoc test.

### 2.7. Compatibility of Regenerated Silk Fibers with Endothelial Cell Cultures: Anti-CD31 Decorated Samples

Another cell line, the Mouse Hemangioendothelioma Endothelial Cells (EOMA), was used to test anti-CD31 decorated fibers. This selection was prompted by the simplified culture procedures of established cell lines compared with primary cultures, and by the expression in this particular line of all critical endothelial markers and functions, including the ability to form tube-like structures [19,20]. EOMA cells were cultured until reaching (70–80%) confluence and suspended with trypsin. The suspended cells were centrifuged and resuspended in vasculogenesis medium (Dulbecco’s modified Eagle medium—DMEM High glucose, 2% FBS, 1% penicillin/streptomycin). 500 μL of a cell suspension (5 × 10^4^ cells/mL) was added to each well, including the polystyrene control, as well as the non-functionalized and anti-CD31 decorated fibers. Cells were incubated at 37 °C, 5% CO_2_ for 24 h before being stained with calcein. Calcein was dissolved in the culture medium at a 1:1000 concentration, and 0.5 mL was added to each well. Cells were incubated with calcein for 30 min at 37 °C and subsequently observed in a fluorescence microscope.

Calcein micrographs were analyzed using ImageJ (v1.54) and MATLAB (v2025a) in order to identify the position of each cell automatically. A position vector was assigned to each cell, and its two coordinates were calculated. The coordinates of the i-cell were used to calculate the values of cosθ_i_ and sinθ_i_ for each cell, which, in turn, were used to determine the average orientation angles, C and S, of the cells in a given cell culture as:

(1) $$C=\frac{\sum \cos\theta_{i}}{N}\;;\;S=\frac{\sum \sin\theta_{i}}{N}\;$$

where N is the total number of cells. From these parameters, it is possible to calculate the Resultant Vector Length, R, as:

(2) $$R=\sqrt{C^{2}+S^{2}}$$

From its definition, a value of R = 0 corresponds to the random orientation of the angles, while a value of R = 1 indicates total alignment.

Additionally, the average direction of a given micrograph, θ_avg_, can be computed from the values of C and S as:

(3) $$\theta_{avg}=\arctan\left(\frac{S}{C}\right)$$

### 2.8. Compatibility of Regenerated Silk Fibers with Organotypic Slices: Methods

Organotypic hippocampal slice cultures were prepared from wildtype C57BL/6-Tg(Thy1-eGFPM) mice of either sex at ages postnatal day (P) 3–6 as previously described [21]. Briefly, freshly dissected 300 µm thick hippocampal slices were transferred onto PTFE semi-porous cell culture inserts of 0.4 μm pore size (Merck/Millipore, Darmstadt, Germany, Cat# PICM0RG50) for long-term air-liquid interface culture of ≥18 DIVs at 35 °C in a humidified atmosphere with 5% CO_2_. The culture medium consisted of 50% (*v*/*v*) minimum essential medium (MEM), 25% (*v*/*v*) basal medium eagle (BME), 25% (*v*/*v*) heat-inactivated normal horse serum (NHS), 2 mM GlutaMAX, 0.65% (*w*/*v*) glucose, 25 mM HEPES buffer solution, 0.1 mg/mL streptomycin, 100 U/mL penicillin and 0.15% (*w*/*v*) bicarbonate. The incubation medium was adjusted to pH 7.30 and renewed 2–3 times a week. All experimental procedures were carried out according to German animal welfare legislation and approved by the appropriate animal welfare committee and the animal welfare office of the University of Freiburg.

To assess the compatibility of regenerated silk fibers with organotypic slices, a single hippocampal slice was plated onto a PTFE semi-porous membrane. A “silk harp” was then immediately placed atop the membrane, leaving the slice in the “window” compartment to interact with the overlapping silk fibers. All slices were maintained under an air-liquid interface and then assessed for structural integrity, gross cytoarchitecture and cytocompatibility. Firstly, we assessed the impact of the harp frame on organotypic hippocampal tissue viability. At DIV 18, all slices were treated with Propidium Iodide (PI; 1 μg/mL; Invitrogen, Waltham, MA, USA, Cat# P3566) for 2 h, then washed with phosphate-buffered saline (PBS; 0.1 M, pH 7.4) and fixed in 4% (*w*/*v*) PFA and 4% (*w*/*v*) sucrose in PBS for 1 h at room temperature. Fixed slices were then washed in PBS and stored at 4 °C prior to immunostaining or staining performed immediately. Positive control slices were treated with N-methyl-D-aspartate (NMDA; 50 μM; Tocris Bioscience, Bristol, UK, Cat# 0114) for 4 h to induce excitotoxicity and then washed and fixed as described. Cell nuclei were stained with DAPI (1:3000; Life Technologies, Carlsbad, CA, USA Cat# 62248) in 0.01 M PBS for 15 min to visualize the cytoarchitecture. Following incubation in DAPI, slices were washed three times in PBS, and the cultures were mounted onto glass slides with anti-fading mounting medium.

Subsequently, we investigated whether silk would cause microglial activation following organotypic hippocampal culture. At DIV 18, slices were immediately fixed as previously described. To visualize microglia, slices were incubated for 1 h at room temperature in blocking solution (10% (*v*/*v*) NGS– goat serum blocking reagent—and 0.5% (*v*/*v*) Triton X-100 —Thermofisher, Waltham, MA, USA— in 0.01 M PBS) to reduce non-specific antibody binding. The slices were then incubated under agitation for 48 h at 4 °C with primary antibody solution (10% NGS and 0.1% Triton X-100 in 0.01 M PBS) containing the guinea pig primary antibody against Iba 1 (1:500, Synaptic Systems, Göttingen, Germany, Cat# 234 308). After 48 h, the slices were washed twice in PBS and incubated overnight at 4 °C with the secondary antibody solution (10% NGS and 0.1% Triton X-100 in 0.01 M PBS) containing the Alexa-labeled secondary antibody (goat anti-guinea pig, Alexa plus 555-labeled secondary antibody; 1:1000; Invitrogen, Waltham, MA, USA, Cat# A32727). The slices were then washed three times with PBS, incubated in DAPI, and mounted as previously described. Confocal images were acquired using a Leica TCS SP8 laser scanning microscope with 20× (NA 0.75; Leica), 40× (NA 1.30; Leica), and 63× (NA 1.40; Leica) oil-immersion objectives.

## 3. Results

### 3.1. Tensile Properties of the Regenerated Silk Fibers

The D3S^TM^ allows varying the diameter of the fibers by using different values of the geometrical and hydrodynamical parameters [17]. The values of the nozzle outlet diameter, dope flow rate and focusing fluid flow rate led to fibers with diameters of approximately 20 μm.

**Figure 3:**
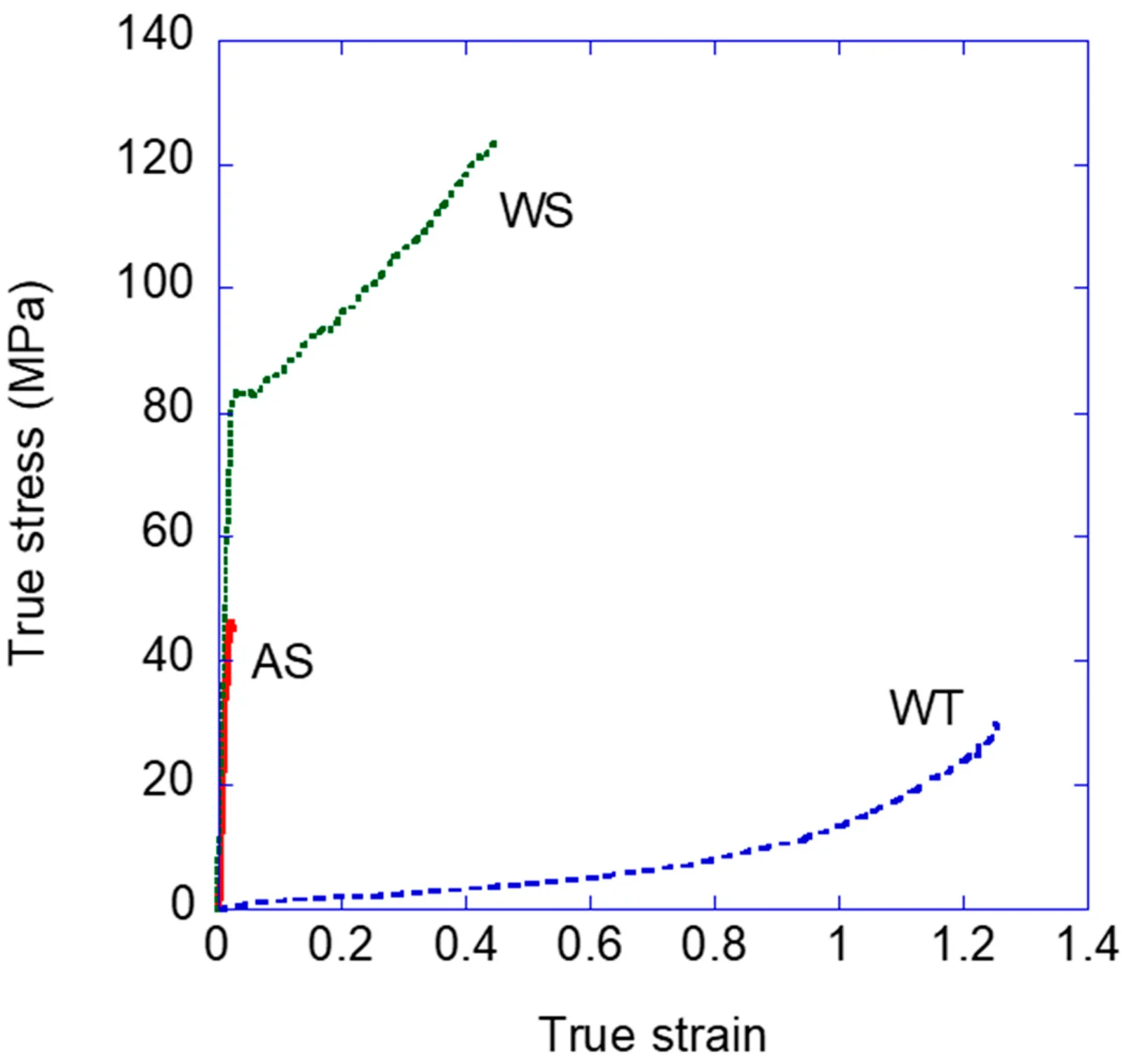
Representative true stress-true strain curve of an as-spun regenerated silk fiber spun with D3S^TM^ tested in air (AS—as spun fiber, red solid line), an as-spun regenerated silk fiber tested in water (WT—fiber tested in water, blue broken line), and a fiber subjected to an 80% wet stretching process (WS—wet stretched fiber, green dotted line).

The *as-spun* (AS) regenerated silk fibers produced via D3S^TM^ typically show a brittle tensile behavior until breaking (Figure 3) with an elastic modulus of E ≈ 3 Gpa and modest values of the tensile strength and strain at breaking that yield a value of work to fracture of W_f_ ≈ 2–5 MJ/m^3^. In contrast, the actual potential of D3S^TM^ regenerated fibers is suggested by their remarkable behavior when tested in water (WT). Although no significant variation is observed in the tensile strength of the fiber, its strain at breaking increases more than two orders of magnitude up to a value of ε_u_ ≈ 1.4 when tested in water. This large value of the strain at breaking allows subjecting the fibers to a wet stretching process [17], in which the fiber is stretched in water up to a predetermined length and subsequently dried. As illustrated in Figure 3, a regenerated fiber subjected to wet stretching (WS) may reach values of σ_u_ ≈ 120 Mpa and ε_u_ ≈ 0.24 that yield a remarkable work to fracture of W_f_ ≈ 30 MJ/m^3^.

### 3.2. Decoration of the Regenerated Fibers with IKVAV and VEGF

**Figure 4:**
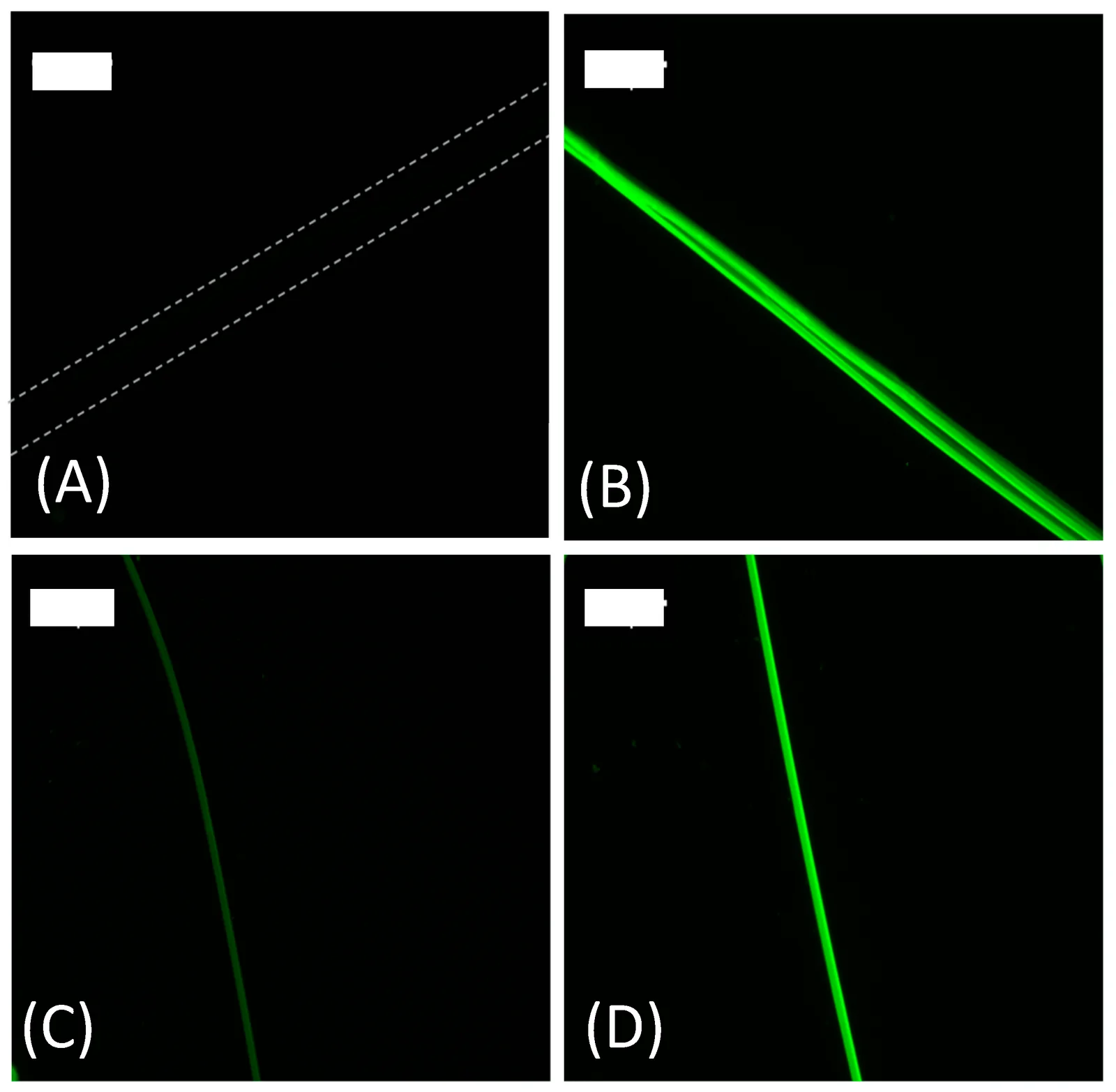
Fluorescence microscopy images of regenerated fibers. (**A**) Incubated with FITC-derivatized IKVAV in the absence of EDC/NHS crosslinker (the silhouette of the fiber is indicated with broken lines), (**B**) Decorated with FITC-derivatized IKVAV through the EDC/NHS crosslinking chemistry, (**C**) Incubated with FITC-derivatized VEGF in the absence of EDC/NHS crosslinker, and (**D**) Decorated with FITC-derivatized VEGF through the EDC/NHS crosslinking chemistry. Scale bar 150 μm.

The effective decoration of the fibers with the IKVAV peptide and with VEGF is verified in Figure 4. Figure 4 compares the fluorescence microscopy images of fibers decorated with both FITC-derivatized bioactive molecules and controls of fibers that were incubated with the respective solutions, either FITC-derivatized IKVAV or VEGF in PBS, but did not contain the EDC/NHS crosslinkers. As observed in the image, except in the case of decorating the fibers with FITC-derivatized molecules, no significant fluorescence was observed in the fibers. This lack of fluorescence is also observed in control fibers not treated with any fluorophore.

### 3.3. Self-Adhesion of Regenerated Silk Fibers

**Figure 5:**
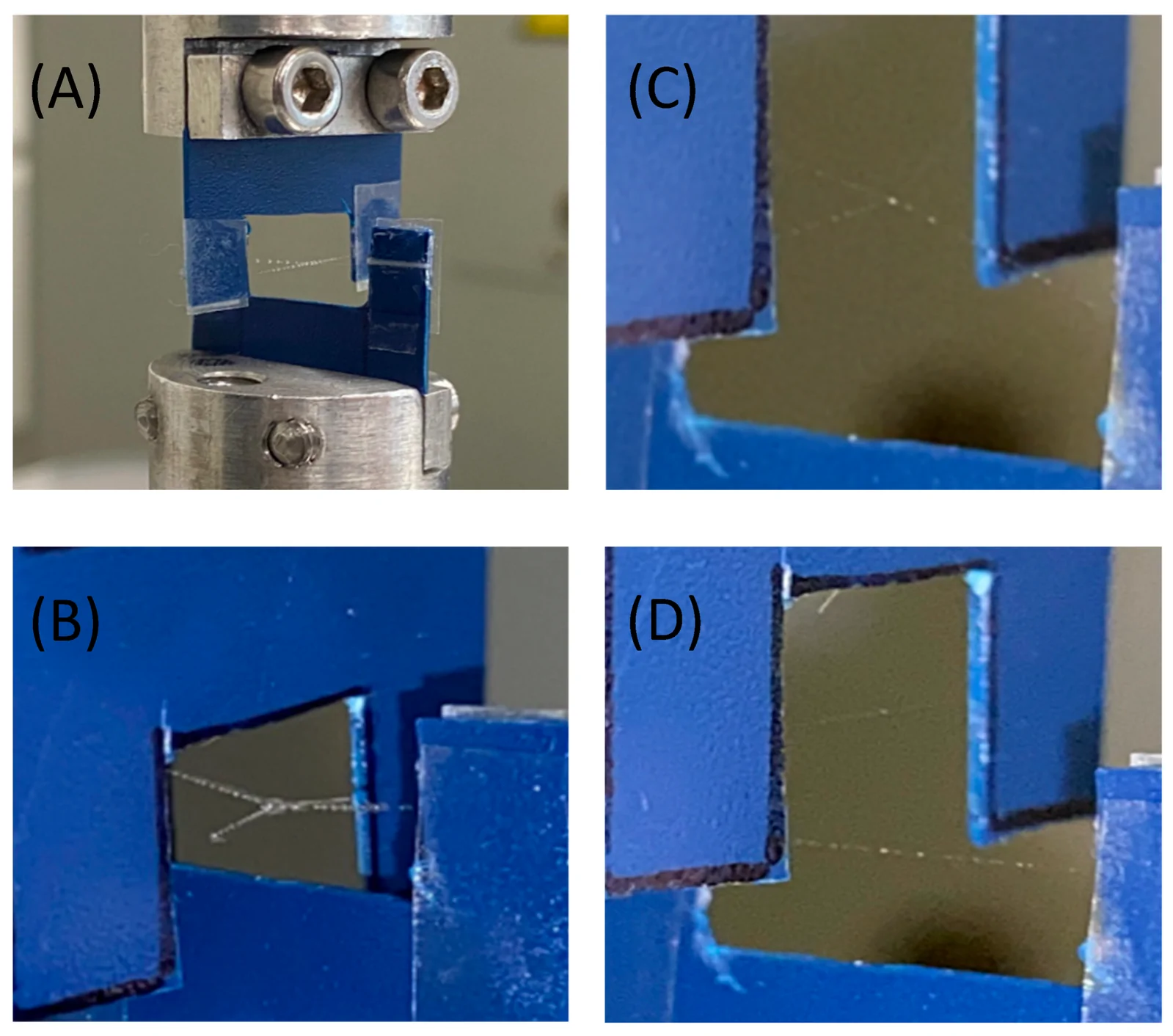
Phases of the self-adhesion test. (**A**) Approximation and contact, (**B**) Pushing, (**C**) Pulling, and (**D**) Detachment.

Figure 5 illustrates the four steps that comprise the self-adhesion test. Firstly, the samples are brought together until they make contact (A) and are pushed to one another up to a predetermined force (B). The fibers are then pulled from one another (C) until getting detached (D). This experimental procedure is used to assess the influence of hydration and drying steps of different durations on the self-adhesion force between regenerated silk fibers.

**Figure 6:**
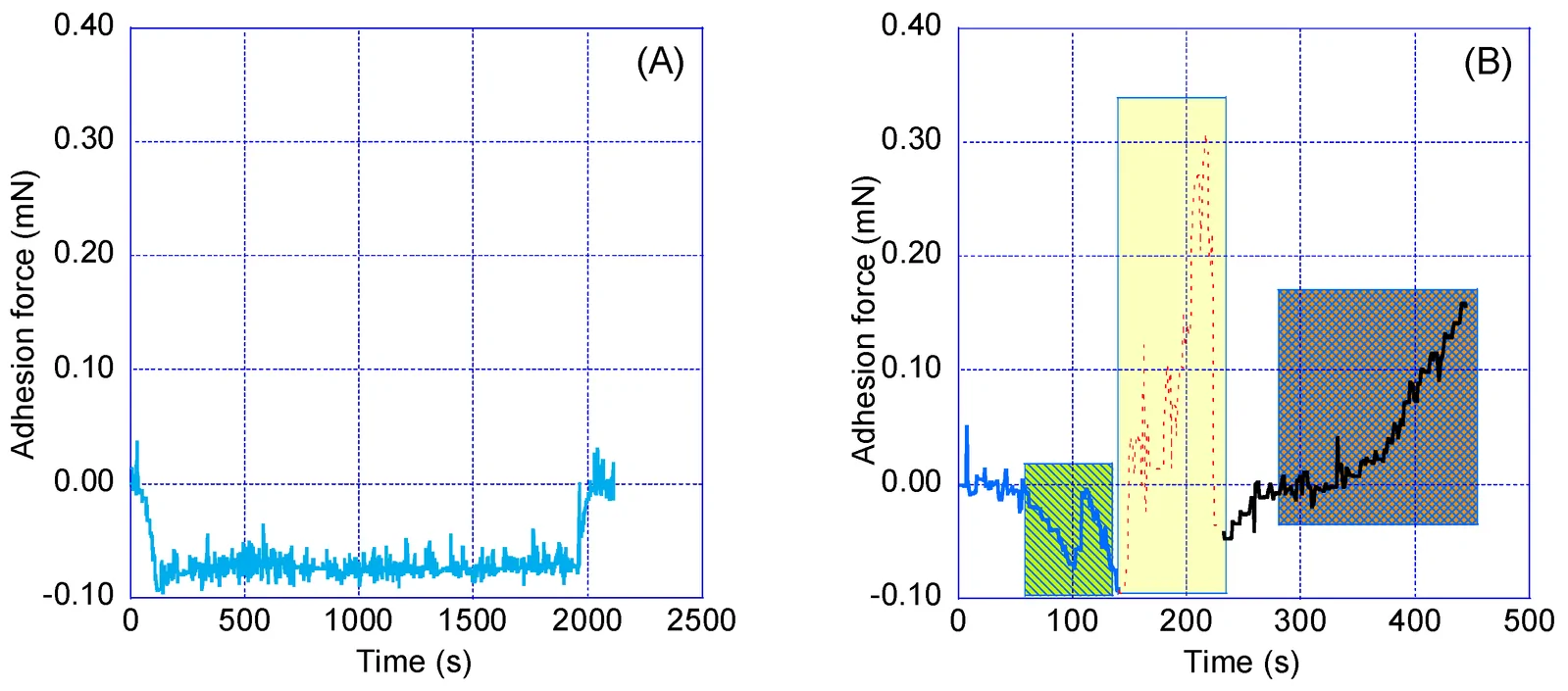
Force vs. time curve of an adhesion test between two regenerated fibers (**A**) not hydrated, (**B**) hydrated for two minutes before the test (100–240 s). Negative values and positive values of force correspond to compression and tensile forces, respectively.

Figure 6A shows the result of a representative self-adhesion test between two silk fibers that were not hydrated at any step of the process. The approximation step begins at t = 0 s and proceeds until a contact force of F = −7 mN is measured. This contact force is maintained for a duration of 1800 s and ends with the beginning of the pulling step. It is observed that the maximum detachment force corresponds to F = 0 mN, which indicates the absence of any self-adhesion between the fibers under these experimental conditions, which do not comprise the hydration of the fibers at any moment. In contrast, Figure 6B illustrates the self-adhesion test of two regenerated silk fibers under hydration conditions. At t = 0 s, the approximation of both fibers begins, and contact is established at t = 60 s. Pushing proceeds until a force of F = −7 mN is reached (green rectangle with slanted segments). Contact is maintained during the hydration step that proceeds for two minutes from t = 100 s to approx. t = 240 s (light brown rectangle). The significant fluctuations of the force during the hydration step are a consequence of the flow of water vapor, which leads to forces within the resolution of the balance. As expected, the fluctuations disappear at the end of the hydration step. Lastly, the pulling step (dark brown rectangle, checkered pattern) starts with a compressive force of F = −5 mN and moves toward positive values of force, indicating the change in regime from compressive to tensile stresses until it reaches a maximum value of F = 16 mN at which the detachment of the fibers is observed. Although these data are to be taken as illustrative, since the full characterization of the self-adhesion effect and of its microscopic mechanism will require a dedicated experimental campaign, a gross estimation of the adhesion stress is obtained in this adhesion test by assuming an average diameter for the fibers of 20 μm. It is thus estimated that the maximum contact area between both fibers must be smaller or equal to A = 400 μm^2^, which yields a value for the adhesion stress of approx. σ_a_ = 40 MPa.

### 3.4. Silk Fiber Scaffolds Induce Highly Aligned Claudin-5 Organization in MEBECs

**Figure 7:**
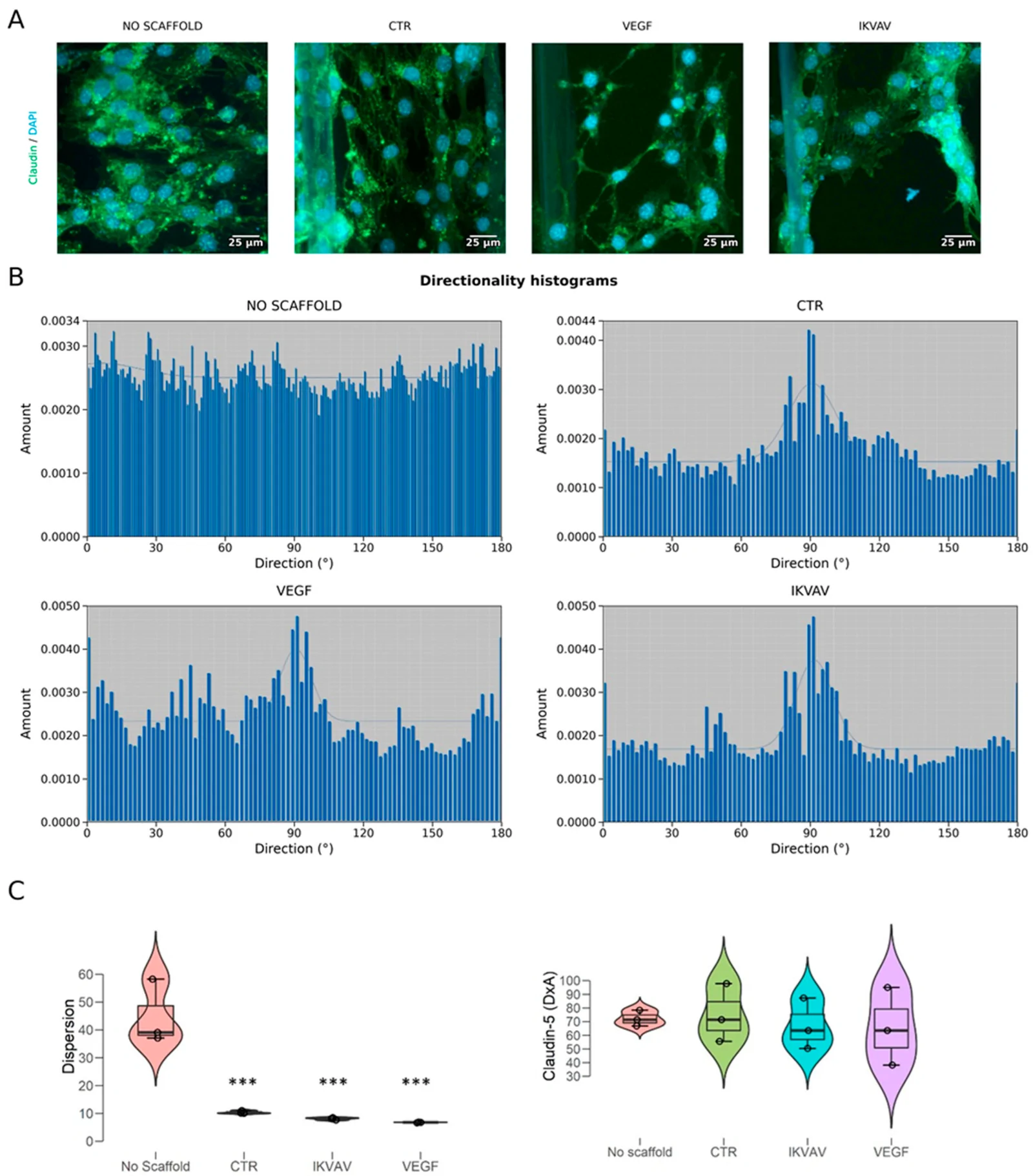
Silk scaffolds promote aligned claudin-5 organization in MEBECs. (**A**) Representative images of claudin-5 immunostaining with DAPI nuclear counterstaining in the no-scaffold, non-functionalized scaffold control (CTR), VEGF, and IKVAV groups. Scale bars = 25 µm in all images. (**B**) Directionality histograms generated in Fiji using the Fourier components method. The *x*-axis represents structural orientation from 0° to 180°, and the *y*-axis reports the corresponding value generated by the directionality analysis. The scaffold-containing conditions show narrow peaks consistent with anisotropic organization, whereas the no-scaffold condition shows a broad distribution. (**C**) Quantification of angular dispersion, expressed in degrees, and claudin-5 integrated fluorescence density, expressed as DxA in arbitrary units (a.u.). Data are presented as mean ± SD. Statistical analysis was performed using one-way ANOVA followed by Tukey’s post hoc test; *** *p* < 0.001 versus the no-scaffold group. No significant differences in angular dispersion were found among CTR, IKVAV, and VEGF, and no significant differences in claudin-5 integrated fluorescence density were found among any of the groups.

To determine the structural impact of biomimetic silk architectures on endothelial monolayer organization, the spatial distribution and alignment of the key tight junction protein, claudin-5, were evaluated in MEBECs at DIV5 (Figure 7). Qualitative immunofluorescence analysis revealed a striking contrast in cellular organization dictated by the physical microenvironment. In the absence of a structural template, claudin-5 boundaries appeared completely disorganized, trace-reconstructing a disordered cell monolayer characterized by random orientations. Conversely, cells cultivated on the structured silk scaffolds formed highly coordinated, elongated cellular cords stretching parallel to the underlying linear topographical features. This morphological alignment was strictly confined within the interstitial spaces between adjacent linear silk fibers.

This contact-guidance phenomenon was further characterized using the Fiji Directionality tool based on Fourier component analysis. The generated directionality histograms revealed distinct topological signatures between the experimental groups. For all structured scaffolds, including the non-functionalized control (CTR), IKVAV, and VEGF decorations, the histograms displayed sharp, narrow, and highly synchronized single peaks centered near 90°, confirming a highly anisotropic distribution of structural orientation angles along the long axis of the silk fibers. Perpendicular small bridges (45°) of cells are also visible in the FFT, but do not determine a change in the preferred orientation. In stark contrast, the no scaffold condition presented a broad, multi-peak, and essentially flat distribution across the entire 0° to 180° spectrum, mathematically highlighting the heightened spatial randomness inherent to unguided environments.

Quantitative fitting of these Fourier profiles validated a profound reduction in angle dispersion for all engineered conditions (Figure 7). The no-scaffold control displayed an exceptionally high angle dispersion of 45° ± 7°. Introducing the scaffolds drove a statistically significant increase in alignment, dropping dispersion values down to 10.4° ± 0.3° for the control (CTR), 8.1° ± 0.3° for the IKVAV functionalization, and 6.77° ± 0.06° for the VEGF functionalization (*** *p* < 0.001 vs. no scaffold). No significant differences in orientation dispersion were observed among the three scaffold groups (CTR, IKVAV, or VEGF). This lack of variance indicates that the physical contact guidance provided by the core linear silk geometry serves as the primary driver of macro-scale cellular alignment, considering the claudin-5 linear redistribution, operating independently of secondary biochemical functionalization.

Although the scaffold architecture markedly altered the spatial organization of claudin-5 staining, it did not produce a statistically significant change in total claudin-5-associated fluorescence. Claudin-5 integrated fluorescence density was 72 ± 3 a.u. in the no-scaffold group, 75 ± 12 a.u. in the non-functionalized scaffold control group (CTR), 67 ± 11 a.u. in the IKVAV group, and 66 ± 16 a.u. in the VEGF group. No significant differences were detected among the experimental groups. These results indicate that the linear regenerated silk fibroin scaffolds provide strong topographical guidance and anisotropic organization of claudin-5-associated structures without detectably increasing or decreasing the overall claudin-5 immunofluorescence signal. This guidance represents a first foundational step toward the development of guided vascular architectures without upregulating or depressing baseline claudin-5 expression.

### 3.5. Compatibility of Regenerated Silk Fibers with Endothelial Cell Cultures: Anti-CD31 Decorated Fibers

**Figure 8:**
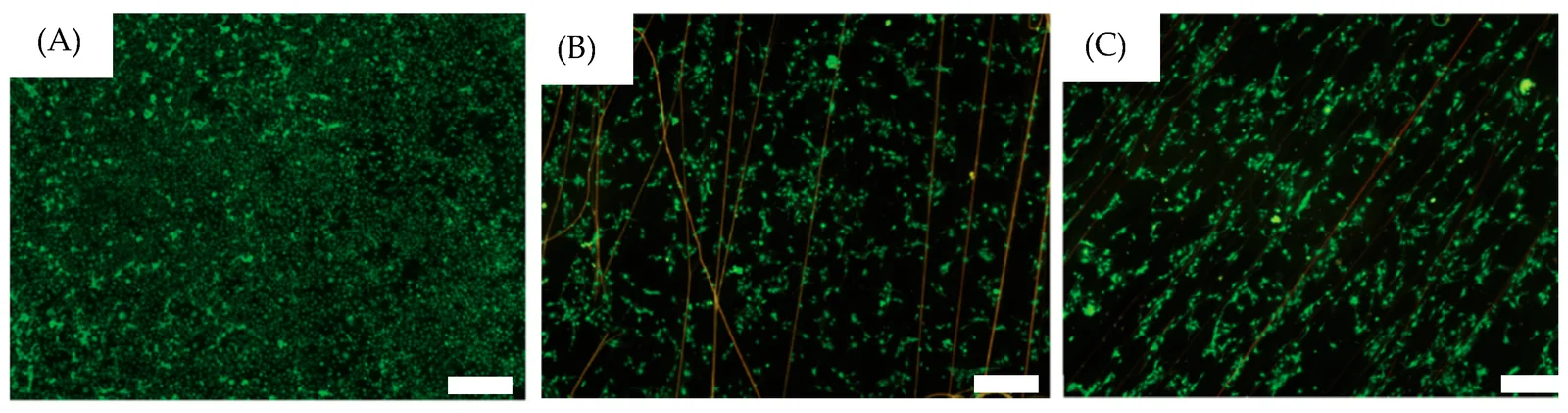
Representative micrographs of EOMA cells cultured in combination with non-functionalized fibers or with decorated fibers. (**A**) Polystyrene control without fibers, (**B**) non-functionalized fibers, (**C**) anti-CD31 decorated fibers (**C**). Cells are identified with calcein staining and observed in a fluorescence microscope. Scale bar 500 μm.

The influence of the anti-CD31 decoration on the organization of endothelial cells was assessed by culturing EOMA cells in the presence of non-functionalized and anti-CD31 decorated fibers. Representative micrographs of cultures at DIV1 are presented in Figure 8, which also includes a control cell culture on polystyrene (A). The usage of the Resultant Vector Length, R, yields a value of R_control_ ≈ 0 for the polystyrene control. R_non-func_ = 0.54 ± 0.04 for the non-functionalized fibers, and R_decorated_ = 0.8 ± 0.1 for the anti-CD31 decorated fibers. These values can be compared with the value of R computed for the fibers, R_fiber_, which yields a value of R_fiber_ = 0.95 ± 0.04. As expected, the absence of geometrical cues in the polystyrene control leads to an essentially isotropic cell distribution, while the presence of the fibers in the culture leads to a significant orientation of the cells. The larger value of R in the anti-CD31 decorated fibers further supports the effect of the decoration in establishing a close connection between the cells and the material. The influence of the fibers on the orientation of the cells is also highlighted from the analysis of the θ parameter since, in this case, a value of Δθ_dec_ = θ_fibers_ − θ_dec_ = 20 ± 10° is obtained for the anti-CD31 decorated samples in contrast with the much larger value of Δθ_non-dec_ θ_fibers_-θ_non-dec_ 71 ± 4° determined for the non-functionalized fibers.

**Figure 9:**
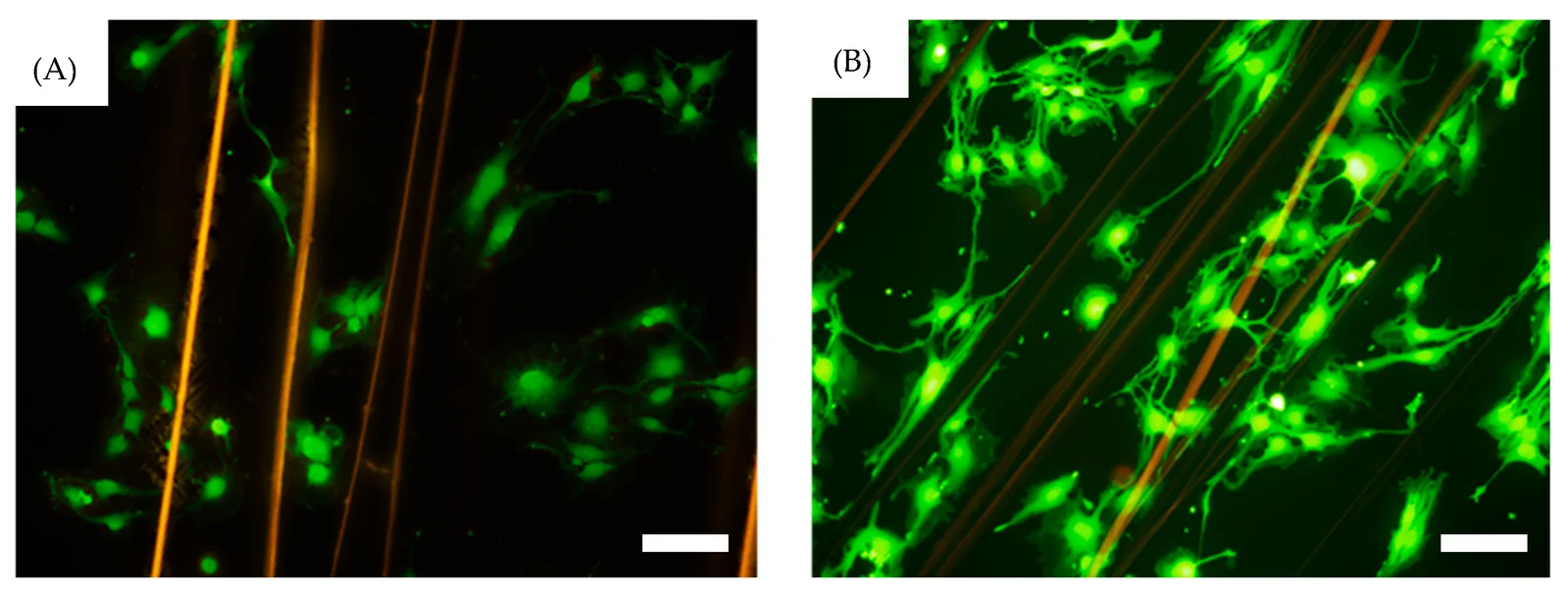
Representative micrographs of endothelial cells cultured on (**A**) control fibers, and (**B**) anti-CD31 antibody decorated fibers. Scale bar 100 μm.

Figure 9 compares the interaction between the EOMA cells and the fibers at higher magnification and allows observing the significant morphological changes induced in the cells through their contact with the anti-CD31 decorated fibers.

### 3.6. Compatibility of Regenerated Silk Fibers with Organotypic Slices

**Figure 10:**
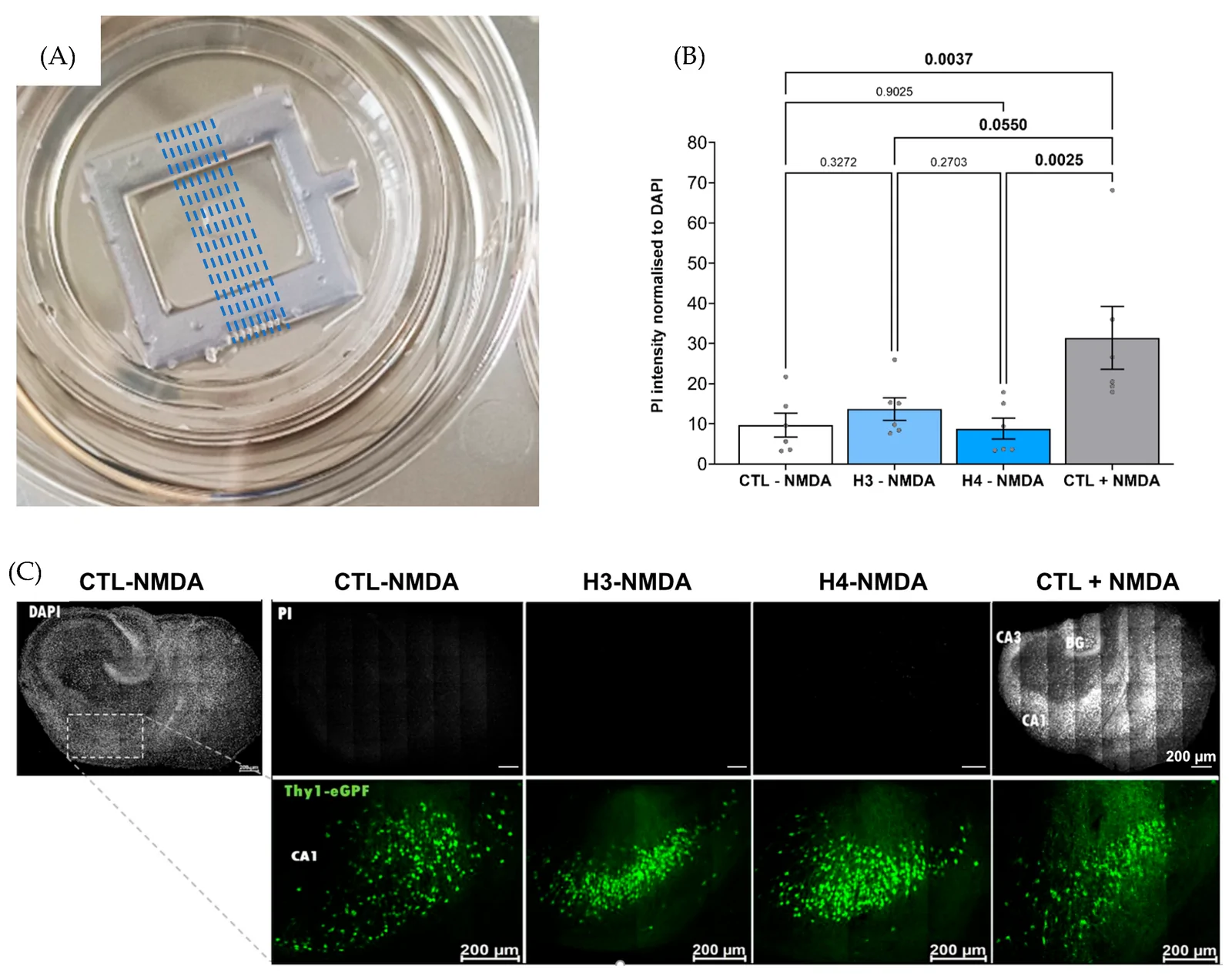
(**A**) Scaffold termed within the THOR framework as “silk harp”, which consists of a set of parallel fibers (indicated by the broken lines in the figure) mounted on a rectangular polymeric frame. The fibers are glued to the frame with biocompatible adhesive. (**B**) Statistical analysis of differences in PI fluorescence intensity showing that there were no significant differences between the CTL–NMDA organotypic hippocampal slices and those cultured either with “silk harps” (*n* = 6 slices/group, Dunn’s multiple comparisons test, *p* > 0.9999). NMDA-treated CTL slices (CTL + NMDA) had a statistically significant increase in PI immunofluorescence compared to the untreated CTL, H3 and H4 groups (*n* = 6 slices/group, Kruskal–Wallis test, *p* = 0.0085). Values represent mean ± SEM. (**C**) Representative images of DAPI (Left image, CTL–NMDA) and PI (Top panel) staining of organotypic hippocampal slices maintained without the use of a silk harp (CTL–NMDA) and slices cultured in the presence of the H3 (H3–NMDA) or the H4 (H4–NMDA) silk harps, and CTL slices that were treated with NMDA (CTL+NMDA). Images were acquired with a confocal microscope using a 20× objective. Scale bar = 200 µm. Thy1-eGFP fluorescence is demonstrated in high-magnification micrographs of the CA1 regions of corresponding groups.

**Figure 11:**
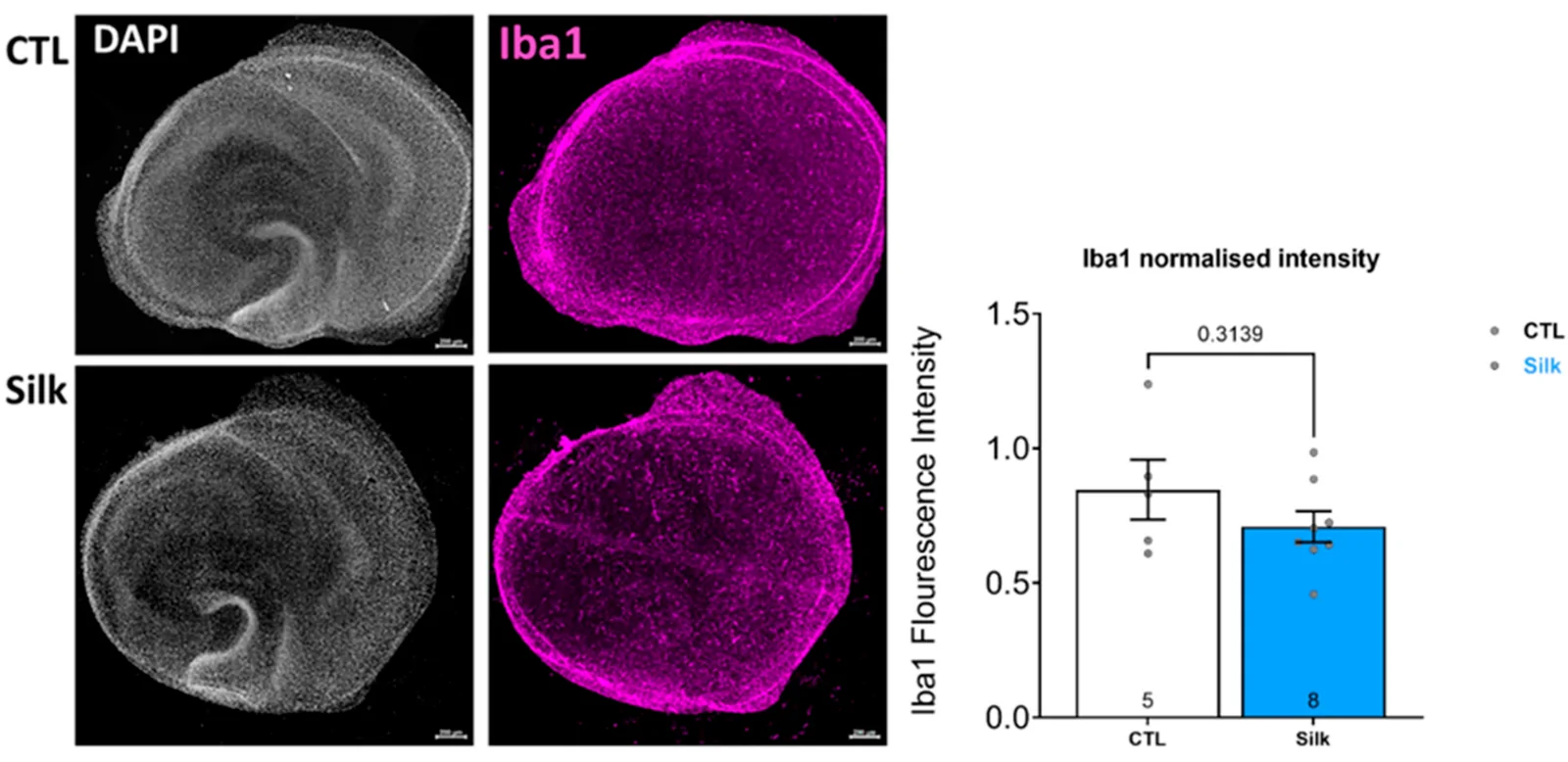
(**Left**) Representative images of DAPI and Iba1 staining of organotypic hippocampal slices cultured in the presence of silk harps. Images were acquired with a confocal microscope using a 40× objective. (**Right**) Statistical analysis of differences in Iba1 fluorescence intensity indicates that there were no significant differences between the CTL and Silk organotypic hippocampal slices (*n* = 5–8 slices/group, Unpaired Student’s *t*-test, *p* = 0.3139). Values represent mean ± SEM.

We investigated the compatibility of regenerated silk fibers in organotypic slices through the use of the “silk harp” approach designed for high-throughput use of non-decorated and functionalized silk fibers as versatile building blocks for biofunctional tissue scaffolds. We assessed the biocompatibility of the silk harp frames on organotypic hippocampal slices using PI and DAPI staining at DIV 18 (Figure 10B,C). Overall, the presence of the “silk harp” frame did not affect the viability of the slices (Figure 10B), and there was no impact on their cytoarchitecture (Figure 10C; Top panel). Moreover, there were no disruptive effects on Thy1-eGFP expression in the CA1 regions of these groups (Figure 10B, below). We investigated the compatibility of silk on the slices using non-decorated “silk harps”, more especially whether culturing with silk would result in microglial activation. At DIV 18, statistical analyses determined that there were no significant differences in Iba1 expression through immunofluorescence intensity measurements (Figure 11), with no evidence of microglial activation, as there were no changes in microglial morphology due to organotypic culture in the presence of silk. Although we identified an interaction of silk with Iba1+ positive cells, there were no changes in morphology of these microglia, suggesting non-reactivity as previously described [22]. In addition, overall structural integrity and slice morphology were maintained in the presence of silk with no significant changes in Thy1-eGFP expression.

## 4. Discussion

The development of the D3S^TM^ process within the framework of the WWT addresses a critical bottleneck in biomaterials engineering: the efficient production of mechanically robust and easy-to-biofunctionalize silk fibroin fibers without the use of toxic organic solvents. By leveraging the natural self-assembly properties of *Bombyx mori* silk fibroin under shear stress and pH variations, the D3S^TM^ platform successfully replicates aspects of natural silkworm extrusion. The as-spun regenerated silk fibers show an elastic modulus (~3 GPa) when tested in air, which is indicative of highly aligned protein chains and a dense network of crystalline β-sheet domains embedded within an amorphous matrix [23,24]. This initial stiffness matches or exceeds traditional regenerated silk formats, which frequently require harsh post-spinning treatments (such as methanol exposure) to induce crystallization. Upon exposure to moisture or in situ hydration, the material exhibits significant changes in its tensile behavior. In particular, it is found that hydrated fibers are bound through intense adhesion forces, requiring a detachment stress of approximately 40 MPa following initial fiber-to-fiber compression. This transition from a rigid, brittle behavior in the dry state to a compliant, highly adhesive behavior when hydrated highlights the plasticizing effect of water molecules on the amorphous regions of the fibroin backbone. Such compliance is highly advantageous for biomedical applications, as it allows the fibers to adapt to the dynamic, wet environments of living tissue without causing mechanical mismatch or structural failure.

Additionally, a central challenge in functionalizing silk scaffolds is ensuring the long-term retention of biochemical cues under physiological conditions [25]. Our comparative fluorescence analysis between physically adsorbed and covalently bound FITC-labeled peptides demonstrates the limitations of simple physical adsorption [26,27]. In this regard, physical adsorption relies heavily on weak electrostatic and hydrophobic interactions, which are easily disrupted during standard washing cycles, leading to a massive burst release of the bioactive moieties. By introducing EDC/NHS crosslinking chemistry or, alternatively, the streptavidin/biotin system, we successfully achieved stable, covalent tethering between the carboxylic acid side chains of the silk fibroin backbone and the amine termini of the functional target sequences (IKVAV and VEGF) or CD31 antibodies [28]. By decoupling these strategies, the WWT demonstrates a dual-action capability: anti-CD31 functionalization handles the physical and spatial recruitment of endothelial networks, while IKVAV/VEGF decoration supplies the long-term, sustained microenvironmental signaling necessary for complex tissue survival and integration. The persistence of the fluorescent signal after rigorous washing protocols confirms that covalent immobilization prevents immediate wash-out. This ensures a localized, sustained presentation of biochemical cues directly to interacting cells, which is essential for guiding complex cellular behaviors like migration, alignment, and angiogenesis. It should be mentioned that, although VEGF has been associated with antimicrobial host defense in specific experimental contexts, this activity was not assessed in the present study. In endothelial cells infected with *Streptococcus pyogenes*, exogenous VEGF has been reported to enhance xenophagy and lysosomal activity, thereby promoting intracellular bacterial clearance [29]; however, no direct inhibition of bacterial growth was observed in cell-free culture. Therefore, the present results should not be interpreted as evidence of antimicrobial activity of the VEGF-functionalized silk fibers. Determining whether covalent immobilization preserves, modifies, or suppresses any potential antimicrobial effect of VEGF will require dedicated microbiological studies. Thus, the biological utility of the functionalized D3S^TM^ fibers was validated across two distinct biological complexity scales: isolated cell lines, including brain-specific endothelial cells, and complex organotypic tissue slices. Endothelial integration is an absolute prerequisite for vascular tissue engineering and BBB scaffold development in vivo. The culture of EOMA endothelial cells alongside primary mouse brain endothelial cells (MEBECs) on VEGF, IKVAV, and anti-CD31-decorated silk fibers resulted in robust cellular attachment, proliferation, and distinct longitudinal alignment along the fiber axes. Achieving structural alignment and phenotypic preservation in highly sensitive primary cells like MEBECs confirms that the combination of topographical cues (the fiber microtopography) and biochemical cues (the immobilized bioactive molecules) acts synergistically to dictate vascular cell behavior [30]. At the organotypic level, the long-term survival of brain slices cultured on the silk fiber harps further illustrates the cytocompatibility and translational potential of this platform. Organotypic slices are notoriously sensitive to substratum toxicity and poor mass transport. The high cell survival rates verified by DAPI/PI, anti-Iba1 and Thy1-eGFP fluorescence demonstrate that the silk harps provide an excellent, non-toxic microenvironment. The open architecture of the fiber array allows for unhindered nutrient and oxygen diffusion while maintaining structural support, mimicking the supportive extracellular matrix networks found in vivo. The robust growth of endothelial cells alongside the long-term survival of organotypic tissue slices on our silk harps validates the excellent cytocompatibility of the WWT platform. This performance mimics successful ex vivo setups where silk matrices have been modified to support delicate microvascular or neural boundaries, proving that functionalized silk can act as a crucial bridge between standard 2D cultures and translationally limited animal models [31]. Taken together, these findings position the functionalized WWT silk platform as a highly versatile candidate for advanced tissue engineering, neural interfaces, and organ-on-a-chip models.

## 5. Conclusions

As originally proposed in the European Pathfinder THOR project, high-performance silk fibers are shown to exhibit a combination of mechanical robustness, self-adhesion, and biofunctionalization capacity that makes them promising building blocks for the development of Web Weaving Technology (WWT). Regenerated silk fibers spun through the Dynamic Dope Destabilization Spinning (D3S^TM^) process can be produced with a variety of geometries, specifically with different diameters, and tensile properties that combine different values of tensile strength and strain at breaking, which, combined, yield values of work to fracture in excess of 30 MJ/m^3^. It is also shown that regenerated fibers are compatible with various crosslinking chemistries, including the EDC/NHS and streptavidin/biotin systems, as well as decorations in terms of bioactive molecules. An original finding of THOR, of significant relevance for the development of the WWT, is the self-adhesion ability of silk fibers. This property allows binding different fibers in order to create the 3D fibrillary structure that makes up the scaffold within the context of WWT. The compatibility of the whole scheme is assessed through cell and organotypic slice cultures, which, in addition, offer some preliminary data on the supporting effect of the fibrillary scaffold on the biological system in vitro. Needless to say, further work will be required to validate long-term stability, vascular functionality, scalability of the 3D assembly, and performance in more complex tissue models, including, most conspicuously, in vivo implantation and assessment of the vascularization of the engineered tissue upon implantation.

## Data Availability

The original data presented in the study are included in Figures throughout the article. Further reasonable inquiries regarding data access can be directed to the corresponding author.
